# Case Cluster of Necrotizing Fasciitis and Cellulitis Associated with Vein Sclerotherapy

**DOI:** 10.3201/eid1401.070250

**Published:** 2008-01

**Authors:** Hiu-Tat Chan, Jillian Low, Lorraine Wilson, Owen C Harris, Allen C Cheng, Eugene Athan

**Affiliations:** * St. John of God Pathology, Geelong, Victoria, Australia; † Barwon Health, Geelong, Victoria, Australia

**Keywords:** Group A Streptococcus, necrotizing fasciitis, sclerotherapy, polidocanol, varicose vein, letter

**To the Editor:** Varicose vein sclerotherapy is a commonly performed cosmetic surgical procedure in which a sclerosing agent is injected into small varicose veins of the leg by using small gauge needles. It is regarded as a minor, safe procedure, usually performed in an office clinic (*1*). We describe a cluster of infections with group A *Streptococcus* spp. associated with throat carriage in a cosmetic surgeon.

In early December 2006, 3 patients were seen over a 10-day period at Geelong Hospital with infections following varicose vein sclerotherapy. All patients had undergone varicose vein sclerotherapy with polidocanol (Laurath-9; Aethoxysklerol, BSN Medical, Mount Waverley, Victoria, Australia) at a clinic of a single cosmetic surgeon. The index patient (patient A) had toxic shock syndrome and necrotizing fasciitis of the treated legs. The 2 other patients (patients C and D) had multifocal cellulitis directly correlating to the injection sites. The time between sclerotherapy and disease onset was 1–2 days.

A case-patient was defined as a patient who had undergone sclerotherapy at the clinic and subsequently had infection directly related to the site of sclerosant injection. Events were dated from the day on which the index patient had her surgical procedure. We reviewed clinic notes and infection control procedures in conjunction with the Department of Human Services of the State Government of Victoria, Australia. Specimens, where available, were collected for culture from patients by the treating clinicians. A throat swab was taken from the cosmetic surgeon. Specimens were transported and cultured by using standard methods.

During the outbreak period, 44 patients had vein sclerotherapy with 3% polidocanol at the cosmetic surgeon’s clinic. In addition to the 3 patients identified on admission to hospital, a fourth patient (patient B) sought treatment from her general practitioner for medical care for a postprocedure infection. All patients had procedures on day 1 or day 7 (Figure); patients A and B were seen consecutively on day 1, and 2 patients were treated between patients C and D on day 7.

**Figure:**
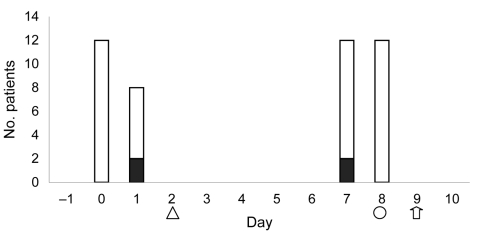
Days of procedures for infected and noninfected patients and their first manifestations of infection. □, uninfected; ■, infected; △, patients A and B seen with infection; Ο, patient C seen with infection; and ↑, patient D seen with infection.

Patient A required surgical debridement, intravenous antimicrobial drugs, intensive care, and hyperbaric oxygen therapy. Intraoperative specimens taken from her during debridement cultured group A *Streptococcus* spp. Patients B, C, and D had cellulitis, but no specimens suitable for microbiologic diagnosis of cellulitis were taken for culture. Patient B was treated with oral antimicrobial agents as an outpatient. Patient C was admitted to hospital for intravenous antimicrobial therapy, and patient D showed no improvement on oral antimicrobial therapy as an outpatient and was subsequently admitted to hospital for intravenous antimicrobial agents.

Group A *Streptococcus* spp. was isolated from a throat swab taken on day 16 from the cosmetic surgeon. He reported no upper respiratory tract infection symptoms before the outbreak. He also reported that antiseptic skin preparation was not routinely used during the procedures; nor were gloves used. However, alcohol hand rubs were used between patients. The surgeon had not changed his infection control procedures recently and had not been aware of any infective complications previously. Environmental surface swabs taken on day 14 from 3 different areas (procedural trolley, surgical spotlight, and examination couch) in the clinic during the assessment yielded no pathogenic organisms. The infection control assessment team noted overall cleaning, disinfection, and hand hygiene to be inadequate.

Decolonization of the surgeon was performed by using rifampin 600 mg daily and amoxicillin 500 mg every 6 hours for 10 days, during which time the surgeon suspended surgical procedures. Recommendations were made regarding infection prevention practices; these were undertaken by the surgeon.

Although soft tissue infection following sclerotherapy may be underreported, large case series have not noted this complication in the past (*2*,*3*); this finding suggests that any soft tissue infection following sclerotherapy should be investigated. These cases highlight the need for vigilance when considering infection control for minor procedures that take place outside of the support of hospital-based infection control services.

Soft tissue infections as complications following varicose vein sclerotherapy appear to be rare (*1*–*3*). The Australian Aethoxysklerol study reported no cellulitis in 16,804 legs injected with the sclerosing agent, and superficial thrombophlebitis occurred at a rate of 0.08% at 2-year review (*2*). Likewise, a multicenter registry with 22 European phlebology clinics reported no cellulitis or necrotizing fasciitis in 12,173 sessions (*3*).

Similarly, surgical site infections with Group A *Streptococcus* spp. are uncommon. A multicenter survey of 72 centers worldwide reported all β-hemolytic *Streptococcus* spp. (including group A and group G) accounted for <5% of infections (*4*), while surveillance in the 1990s by Centers for Disease Control and Prevention reported <1% of all surgical wound infections was caused by group A *Streptococcus* spp. (*5*). A Canadian study reported invasive group A *Streptococcus* infections following surgery in 1.1 cases per 100,000 admissions (*6*). Outbreaks have been infrequently described (*5*,*7*–*10*), and sources of colonization range from throat to anus and vagina.
